# Recovering task fMRI signals from highly under-sampled data with low-rank and temporal subspace constraints

**DOI:** 10.1016/j.neuroimage.2018.02.062

**Published:** 2018-07-01

**Authors:** Mark Chiew, Nadine N. Graedel, Karla L. Miller

**Affiliations:** Wellcome Centre for Integrative Neuroimaging, FMRIB, Nuffield Department of Clinical Neurosciences, University of Oxford, United Kingdom

## Abstract

Recent developments in highly accelerated fMRI data acquisition have employed low-rank and/or sparsity constraints for image reconstruction, as an alternative to conventional, time-independent parallel imaging. When under-sampling factors are high or the signals of interest are low-variance, however, functional data recovery can be poor or incomplete. We introduce a method for improving reconstruction fidelity using external constraints, like an experimental design matrix, to partially orient the estimated fMRI temporal subspace. Combining these external constraints with low-rank constraints introduces a new image reconstruction model that is analogous to using a mixture of subspace-decomposition (PCA/ICA) and regression (GLM) models in fMRI analysis.

We show that this approach improves fMRI reconstruction quality in simulations and experimental data, focusing on the model problem of detecting subtle 1-s latency shifts between brain regions in a block-design task-fMRI experiment. Successful latency discrimination is shown at acceleration factors up to R = 16 in a radial-Cartesian acquisition. We show that this approach works with approximate, or not perfectly informative constraints, where the derived benefit is commensurate with the information content contained in the constraints. The proposed method extends low-rank approximation methods for under-sampled fMRI data acquisition by leveraging knowledge of expected task-based variance in the data, enabling improvements in the speed and efficiency of fMRI data acquisition without the loss of subtle features.

## Highlights

• A method for including task-fMRI design information in image reconstruction is proposed.
• This approach combines low-rank and temporal subspace constraints.
• Improved fidelity of recovered functional information in highly accelerated imaging.

## Introduction

The need to reconstruct fMRI data from under-sampled image acquisition arises in a number of different contexts, to improve temporal or spatial characteristics of the image data, or to reduce artefacts. Improving temporal sampling can increase temporal degrees of freedom for statistical benefit, provide dimensionality necessary for temporal independent component analyses (ICA) (Smith et al., 2012), aid detection and modelling of subtle features of the hemodynamic response (Buxton et al., 2004), mapping regional differences in BOLD latency (Chang et al., 2008), or increasing sensitivity to fast event related experimental designs (Buckner, 1998). Alternatively, under-sampling can enable higher achievable spatial resolutions, facilitating applications such as layer specific fMRI (Goense et al., 2012). In these cases, accelerated imaging also benefits from a reduced impact of artefacts such as physiological noise, which are easier to remove when unaliased, or a reduced effect of longer echo-train artefacts (e.g. distortion, blurring).

While simultaneous multi-slice imaging has emerged as a popular successor to multi-slice EPI, in recent years, a number of different strategies have been proposed for accelerating fMRI data acquisition, not solely dependent on coil-sensitivity encoding. Some examples that leverage compressible representations of fMRI data in some way include compressed sensing (CS) using spatial wavelet or temporal spectral sparsity (Jung and Ye, 2009; Jeromin et al., 2012; Holland et al., 2013; Zong et al., 2014), partially separable function (PS) modelling (Liang, 2007; Lam et al., 2013; Nguyen and Glover, 2014), low-rank modelling (LR) (Chiew et al., 2015; Chiew et al., 2016), and most recently low-rank and sparse decompositions (L + S) (Singh et al., 2015; Petrov et al., 2017; Aggarwal et al., 2017; Weizman et al., 2017). With the exception of the use of spatial wavelet CS, all these methods move away from time-independent reconstruction of 3D volumes, leveraging temporal structure in the fMRI data, as they effectively seek to fit reconstruction models with fewer free parameters to enable reconstruction in the presence of under-sampling.

While CS relies on explicit knowledge of a sparsifying basis or transform domain, and PS relies on *a priori* knowledge of the data’s complete temporal subspace, the advantage of LR models is that they require only that a low-rank representation exists, and no knowledge of the specific characteristics of these spatial and temporal subspaces is required ahead of time. In one sense, the L + S approach improves the robustness of low-rank subspace estimation (i.e. principal component analysis, PCA) by additionally estimating sparse outliers (Candes et al., 2011). The L + S method has also been used in the opposite sense, by using the L component to regularize sparse modelling of the data (Otazo et al., 2015a), although some approaches have proposed interpretations where both the low-rank and sparse components are of functional importance (Weizman et al., 2017).

Most commonly, temporal frequency is used as the sparse domain in CS or L + S reconstructions, via the Fourier transform. However, this requires strong assumptions about the smoothness or periodicity of the signals of interest (Lustig et al., 2006). Event-related and resting-state fMRI, for example, do not exhibit the same kinds of temporal structure, and sparsity constraints on the temporal spectra can bias the data considerably. Here, we propose a different approach to incorporating *a priori* temporal information in an under-sampled fMRI image reconstruction problem, which performs a constrained LR reconstruction in which the temporal subspace of the data is *partially* fixed by the given information. In a sense, this can be seen as a variation of the L + S approach, where the S-component is sparsified by a transform defined by the specific temporal constraint (and not some generic basis).

The proposed method has a meaningful interpretation in the context of fMRI analysis models. Joint PCA-ICA reconstruction has been remarkably successful in analysing fMRI data because these signals lie in subspaces of relatively low dimensionality (Beckmann and Smith, 2004). Moreover, even in task fMRI, a substantial fraction of the variance is not known *a priori*, including the presence of physiological noise, deviation of true activity from the expected task time-course, and the presence of non-specific neuronal fluctuations. Data-driven approaches can capture these signals more comprehensively than pre-specified models. Nevertheless, *a priori* knowledge of fMRI signals, such as the task or confound regressors used in general linear modelling (GLM) (Friston et al., 1995), could provide greater sensitivity in detecting subtle sources of signal variance that are not captured by data-driven approaches like PCA. In this context, the proposed approach can be thought of as a PCA/GLM hybrid model, where LR modelling is used to capture the subspace of fMRI signals (PCA), but with an additional constraint based on known information about the signal’s time evolution (GLM). In other words, we fit what is known about the data (GLM), and let the remaining signal variance be modelled by a low-dimensional (PCA) to constrain the highly under-determined image reconstruction problem.

In this paper we show, through retrospective under-sampling simulations and experiments, the effectiveness of the proposed approach in recovering spatio-temporal BOLD information at high under-sampling factors, when the known or expected experimental BOLD signal modulations are available *a priori*. As an extension of our previous work, we refer to this method as “constrained k-t FASTER”. While this paper focuses on demonstrating improved extraction of subtle latencies in block-design task-fMRI, this approach can be used to more generally leverage any *a priori* knowledge of signal dynamics, such as those derived from measures extrinsic to the MRI sampling procedure.

## Methods

### Reconstruction algorithms

In this paper, our reconstruction models the fMRI data as a space-time matrix $M=UV^{*}$, where U is an n×r matrix of spatial components (r column vectors of spatial maps with n voxels each), V is an t×r matrix of temporal components (r column vectors of time-courses with t points), and ^∗^ denotes the conjugate transpose. In this decomposition, the components in U are weighted by the signal energy, while the components in V are normalized. The resulting product M is an n×t space-time matrix (with images as columns, and time-courses as rows), corresponding to the 4D datasets common to fMRI.

To solve the under-sampled imaging problem, the LR reconstruction is formulated as a non-convex, rank-constrained optimization problem using a fixed, low rank input, which we call k-t FASTER (Chiew et al., 2015). We use a non-convex approach that combines hard thresholding (Blumensath, 2011) with matrix shrinkage (Goldfarb and Ma, 2011), that we have determined to work well for data with fMRI characteristics, particularly with LR models with rank $∼10^{1}$, whereas conventional low-rank models typically operate in a regime where rank $∼10^{0}$ (Otazo et al., 2015b).

**Fig. 1:**
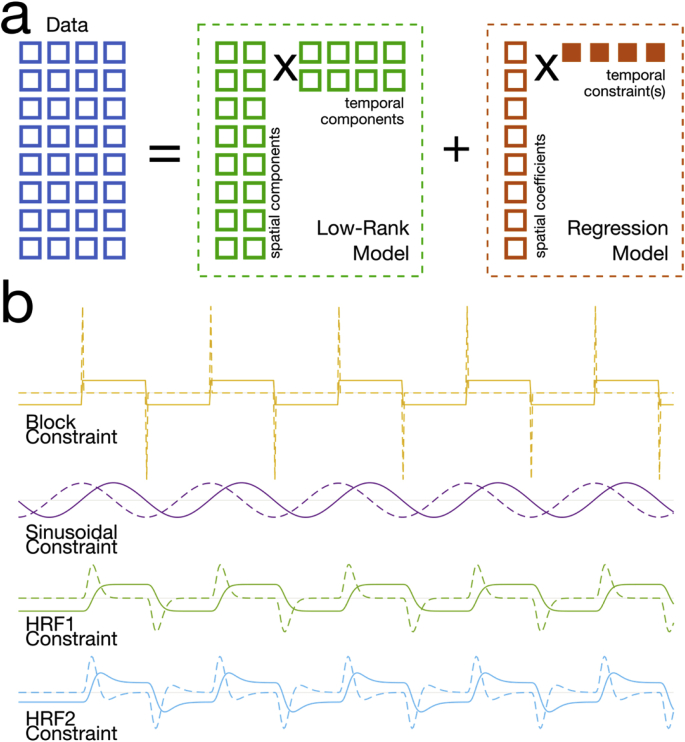
(a) Schematic of the model used to perform image reconstruction. Here, the space-time dataset is partitioned into two parts: (orange) a regression model that fits spatial components to one or more input temporal constraints, and (green) a low-rank model that fits a fixed, and relatively small number of spatial and temporal components to the remaining variance in the measured data. The filled-in boxes represent information that is known *a priori*. (b) Examples of the temporal constraints used here, including block, sinusoid and HRF-convolved waveforms (solid), and their temporal derivatives (dashed).

Our constrained k-t FASTER reconstruction asymptotically solves the following problem:

(1) $$\min_{X_{r},U_{c}}\;\frac{1}{2}{\left|\left|E\left(X_{r}+U_{c}V_{c}\prime \right)-d\right|\right|}_{2}^{2}+\lambda_{r}{\left|\left|X_{r}\right|\right|}_{*}$$

where E is the measurement encoding operator, which encompasses both k-space sampling (which can be non-uniform) and coil-sensitivity encoding, $X_{r}$ is a rank r matrix estimate, $U_{c}$ is the set of spatial coefficients associated with the known temporal constraint in $V_{c}$ (typically demeaned), d is the sampled data, and $∥\cdot {∥}_{*}$ denotes the nuclear norm, or sum of singular values. In the context of more familiar fMRI analysis techniques, $X_{r}$ and $U_{c}$ correspond to the low-rank PCA model and GLM spatial regression coefficients, respectively. In words, the problem can be described as solving for $X_{r}$ and $U_{c}$, such that they are consistent with the measured data (first term above), and additionally that $X_{r}$ has rank r with a minimal nuclear norm (second term above). Here, the scalar $\lambda_{r}$ is defined implicitly by the choice of r, and is related to the soft shrinkage applied to the matrix singular values every iteration (Algorithm 1). A schematic of this reconstruction can be seen in Fig. 1a, and some examples of potential temporal constraints $V_{c}$ are shown in Fig. 1b.

To solve this, we can employ the following constrained iterative hard thresholding and matrix shrinkage (IHTMS) procedure, iterated until convergence or for a maximum number of iterations (Algorithm 1):

Algorithm 1 **Constrained Iterative Hard Thresholding with Matrix Shrinkage. The shrink step requires a singular value decomposition, and effectively finds a rank-truncated representation of the input, similar to a PCA.**

Initialize $M^{0}$ (e.g., as zeros), step size t, rank r, and shrinkage factor τ.

Loop until converged:

$$Y^{\left(i+1\right)}=M^{\left(i\right)}+tE^{*}\left(d-EM^{\left(i\right)}\right)$$

$$U^{\left(i+1\right)}=Y^{\left(i+1\right)}V_{c}{\left(V_{c}^{*}V_{c}\right)}^{-1}$$

$$X_{r}^{\left(i+1\right)}=shrink_{r,\;\tau }\left(Y^{\left(i+1\right)}-U^{\left(i+1\right)}V_{c}^{*}\right)$$

$$M^{\left(i+1\right)}=X_{r}^{\left(i+1\right)}+U_{c}^{\left(i+1\right)}V_{c}^{*}$$

where

$$shrink_{r,\;\tau }\left(X\right):\text{max}\left(diag{\left(s_{j}-\;\tau s_{r+1}\right)}_{1\leq j\leq r},0\right)$$

and $s_{j}$ are the singular values of X.

Alt-text: Algorithm 1

Here, the encoding operator E and its adjoint $E^{*}$ perform non-uniform FFT (and adjoint non-uniform FFT) using the NUFFT (Fessler and Sutton, 2003). Density compensation weights for the non-uniform k-space sampling were generated using a fixed point algorithm (Pipe and Menon, 1999). The operator also performs voxel-wise multiplication of images onto coil sensitivities, and sums the coil images weighted by their conjugate sensitivities as an adjoint operation (Roemer et al., 1990). The shrinkage operation shrinks the first r singular values to generate a nuclear-norm minimized rank r matrix $X_{r}$.

In essence, this approach iteratively estimates the data by first fitting the “GLM” coefficients associated with the temporal constraint, and then identifying a low-rank matrix to explain the remaining variance. The process partitions the row-space (temporal subspace) into orthogonal subspaces using the Gram-Schmidt procedure, such that the GLM temporal subspace is normal to the PCA temporal subspace. Unless the temporal constraints happen to be identically eigenvectors of the data, however, the spatial dimensions (column spaces) will not in general be orthogonal. When no constraint is applied, the reconstruction is identical to the previously report k-t FASTER method using radial-Cartesian sampling (Chiew et al., 2016).

Using techniques from accelerated gradient methods (Nesterov 1983, Beck and Teboulle, 2009), we can also significantly speed up the convergence of the algorithm by adding some momentum to the iterative procedure (see Supplementary Data). All results from the constrained k-t FASTER reconstruction were produced using this accelerated algorithm. Reconstruction code, implemented in MATLAB can be found at http://users.fmrib.ox.ac.uk/∼mchiew/research/.

This procedure depends on forms of sampling incoherence in two ways, one for the GLM fit, and one for the low-rank residual estimation. In the latter case, incoherence requirements are the same as for any low-rank matrix completion problem, namely that the singular vectors not be too sparse in the sampling domain (Candes and Tao, 2010). In the former case, however, we also have an interaction between the temporal aliasing defined by the sampling point-spread function (PSF). As it is impossible to distinguish between aliased signal energy and true signal, sampling incoherence is crucial for minimizing unwanted contributions from aliased signals.

To illustrate the generality of this approach, we also show that this partially constrained subspace framework can be extended to standard convex low-rank matrix recovery problems, which solve:

(2) $$\min_{X_{r},U_{c}}\;\frac{1}{2}{\left|\left|E\left(X_{r}+U_{c}V_{c}\prime \right)-d\right|\right|}_{2}^{2}+\lambda_{SVT}{\left|\left|X_{r}\right|\right|}_{*}$$

Using approaches such as iterative singular value soft thresholding (SVT) (Cai et al., 2010; Candes et al., 2013), a formulation which can be found in most L + S reconstruction approaches (Otazo et al., 2015b).

The primary difference between Eqs. (1), (2) is that a fixed $\lambda_{SVT}$ is chosen in the SVT algorithm (Appendix A) that thresholds singular values based on their amplitude, leaving the actual output rank only implicitly constrained. The problem of selecting an appropriate $\lambda_{SVT}$ parameter is similar to the dimensionality selection of a PCA or hard thresholding problem, where the rank constraints need to be large enough to encompass the range of functional variability, but in this case also small enough to effectively constrain the reconstruction.

Finally, we also compare our constrained k-t FASTER reconstructions to conventional CS and L + S reconstructions using the temporal frequency domain as the sparse regularizer:

(3) $$\underset{X}{\min\;}\frac{1}{2}{\left|\left|EX-d\right|\right|}_{2}^{2}+\lambda_{CS}{\left|\left|F_{t}X\right|\right|}_{1}$$

(4) $$\underset{L,S}{\min\;}\frac{1}{2}{\left|\left|E\left(L+S\right)-d\right|\right|}_{2}^{2}+\lambda_{L}{\left|\left|L\right|\right|}_{*}+\lambda_{S}{\left|\left|F_{t}S\right|\right|}_{1}$$

Here $F_{t}$ denotes the Fourier transform along the temporal dimension, $\lambda_{CS}$, $\lambda_{S}$ are the parameters for the sparsity constraint, and $\lambda_{L}$ weights the low-rank constraint. The CS problem (Eq. (3)) is solved using the FISTA (Beck and Teboulle, 2009) approach (Appendix B), and the L + S problem is solved using the approach described in (Otazo et al., 2015b).

### Simulations

To assess the performance of the proposed constrained reconstruction framework, we used a 2D simulation of a digital phantom with realistic noise properties which we retrospectively under-sampled using a perturbed golden-angle radial sampling scheme (Winkelmann et al., 2007; Chiew and Miller, 2016) at R = 8 (i.e., 8 projections per 64 × 64 image time-point). While in general, radial sampling is less efficient than equivalent Cartesian sampling (Scheffler and Hennig, 1998), so that the actual under-sampling factors are πR/2 (e.g. 12.57 at R = 8), all acceleration factors here are quoted relative to equivalent Cartesian sampling.

**Fig. 2:**
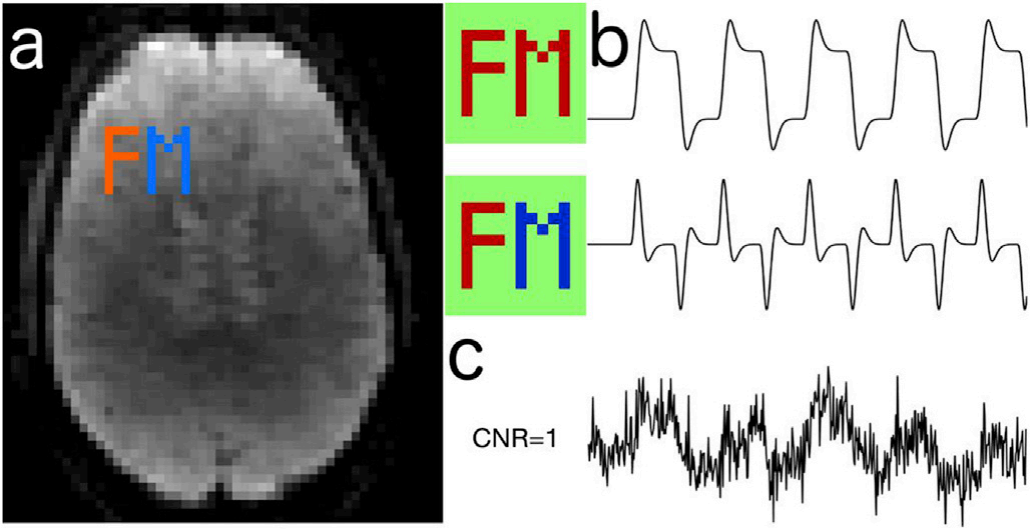
The simulation setup: (a) Two ROIs, the letters “F” and “M” are overlaid on a background brain image with realistic temporal fluctuations, along with additive Gaussian white noise. (b) The signals of interest correspond to a 5-epoch block design task, common to both ROIs (top row), but with a relative latency of 1 s between ROIS (bottom row). The “F” ROI (positive lag) leading the “M” ROI (negative lag), where the colour maps correspond to the amplitude of the timecourse in each pixel. (c) An example time-course from the “F” ROI, showing the CNR of an equivalent fully-sampled dataset.

The simulation (Fig. 2) consisted of two regions of interest (ROIs) that contained the same 5-block off-on BOLD signal variation, generated from a haemodynamic response (HRF)-convolved boxcar waveform. However, a relative lag of 1 s between the ROIs was introduced as a subtle manipulation, to introduce a low-variance functional component. This latency manipulation was not used for any causal inference.

The simulated shot TR was 75 ms, resulting in a reconstructed volume TR of 600 ms over a 5 min simulated duration. Both additive complex Gaussian white noise, and realistic physiological fluctuations extracted from real data were included, and 4 virtual coils with a diagonal noise covariance matrix were used. Under these conditions, fully-sampled data with a sum-of-squares reconstruction would produce BOLD signals with a low contrast-to-noise ratio (CNR) of approximately 1 (Fig. 2c). To assess the variability in parameter estimates, each simulation was repeated 10 times with different sampling patterns and additive Gaussian white noise instances.

Several different temporal constraints and their temporal derivatives, accounting for differing degrees or accuracy of prior knowledge, were used in the assessment of the proposed constrained reconstruction (Fig. 1b). A “block” constraint represented the coarsest signal model, and corresponded to the block design waveform without any HRF convolution. A smoother “sinusoidal” constraint was also generated from a pure sinusoid at the task frequency (1/60 Hz). Lastly, two different HRF models based on the block timing were used to generate more realistic signal models: “HRF1” used a Γ(6,1) Gamma model and “HRF2” used a double Gamma model Γ(6,1)−0.6⋅Γ(5,2) to include a post-stimulus undershoot, with HRF2 used to generate the simulated data.

Reconstructions with the proposed approach used a total rank constraint of 16, with 2 of those components corresponding to an input temporal constraint. A step size of 0.5 and τ=0.1 were used for all cases. For reconstructions using the SVT, CS and L + S approaches, the parameters were tuned for the best case, by post-hoc selection of the optimal values with knowledge of the ground truth. This resulted in $\lambda_{SVT}=1.1\times 10^{-3}$, $\lambda_{CS}=1.65\times 10^{-5}$, $\lambda_{L}=1.8\times 10^{-3}$, and $\lambda_{S}=8.86\times 10^{-6}$ relative to the 2-norm of the data. All methods were implemented with a constant step size of 0.5, and all algorithms were run for 25 iterations, or until the difference between successive estimates was less than $10^{-4}$.

### Experiments

Data were collected on three healthy volunteers, using a block-design visually cued finger-tapping task, at 3 T (Prisma, Siemens Healthineers, Erlangen Germany ) in accordance with local ethics. All data were acquired using a hybrid radial-Cartesian “TURBINE” sampling strategy (Chiew et al., 2016, Graedel et al. 2017) using a golden angle sampling scheme with 5° random pertubations (Chiew and Miller, 2016). In all cases, an additional parallel imaging acceleration factor of R = 2 was applied along the Cartesian z-direction to ensure optimal TEs for BOLD contrast, and reconstructed prior to and independently of the reconstruction in the radial direction using GRAPPA (Griswold et al., 2002).

One subject was scanned using a 2 mm isotropic functional imaging protocol, performing finger tapping with and without a 1-s delay in the left hand relative to the right. The data were acquired at TE = 29 ms, TR = 50 ms, with whole brain coverage, and reconstructed using 10 radial projections for a volume TR (and output temporal resolution) of 500 ms. This corresponded to a radial acceleration factor of R = 10 (10 projections, 100 × 100 matrix), reconstructed with 8 virtual coils (from 32 physical channels) after using an SVD-based coil compression. The same data was also reconstructed at a spatial resolution of 4 mm, for a lower effective acceleration factor of R = 5 (10 projections, 50 × 50 matrix).

To explore higher acceleration and spatial resolution, two subjects were also scanned under different 1.5 mm protocols using the same latency task at TE = 30 ms, with TR = 60 and 75 ms respectively, differing only in TR and axial volume coverage. Both were reconstructed at a volume TR = 600 ms with 8 virtual coils after compression, with the former at R = 12.8 (10 projections, 128 × 128 matrix) and the latter at R = 16 (8 projections, 128 × 128 matrix).

All data were reconstructed using an HRF1-style convolved Gamma model constraint along with its temporal derivative. Rank constraints of 16 were used, with all reconstruction parameters identical to those used in the simulations. All the experimental datasets were also reconstructed using CS with temporal sparsity constraints, using $\lambda_{CS}$ values that were chosen *post-hoc* as the best values given the output metrics and qualitative inspection. This resulted in $\lambda_{CS}=1.80\times 10^{-5},\;7.92\times 10^{-6}$ for the 2 mm/4 mm data, and $\lambda_{CS}=1.59\times 10^{-5}$, $2.34\times 10^{-5}$ for the 1.5 mm data relative to the data norm.

### Statistical quantification of parametric maps

To ensure robust statistical parametric mapping, the quantification procedure performed conventional parametric estimation using the reconstructed data (e.g. t-statistics), and relied on Gaussian-Gamma mixture modelling (Beckmann and Smith, 2004; Feinberg et al., 2010) across the statistics from all voxels to derive corrected statistical distributions that enable valid inference. We employ a 3-distribution model, with a central Gaussian for the majority null-distributed voxels, and Gamma distributions that fit the positive and negative activation tails.

In all the data, the HRF1 model and its temporal derivative were used as the regression design matrix, and all data were magnitude transformed and linearly detrended prior to statistical processing. As the latency effect is only meaningful when a signal is present, z-statistic images for the lag are masked by the main task effect (at |z|>3), which generates an effective “*and”* parametric contrast.

### Latency estimation

Using a first order linear approximation to small shifts in signals, we can model small lags Δt:

$$s\left(t+\Delta t\right)\approx s\left(t\right)+\Delta t\cdot s^{\prime }\left(t\right)$$

where $s^{'}\left(t\right)$ denotes the temporal derivative. Comparing this to the regression model:

$$y\left(t\right)=\alpha \cdot s\left(t\right)+\beta \cdot s^{'}\left(t\right)$$

it is apparent that the lag Δt can be estimated as the coefficient of the derivative term, relative to the coefficient on the signal term (Δt=β/α) (Henson et al., 2002). To assess the relative latencies between left and right sensorimotor cortices (L-SMC, R-SMC), ROIs based on the z-statistics from the main task (defined by an average latency offset) were generated for both L- and R-SMC, based on a |z|>3 criteria, limited to the sensorimotor region, followed by a 1-voxel dilation. Given the relatively small number of voxels in each ROI, and visible non-Gaussianity of the distribution of the Δt metric, we performed planned non-parametric Wilcoxon rank-sum tests to assess the significance of any difference between the Δt estimates from each voxel in the respective L and R-ROIs, assessed at p < 0.05. Given the self-paced latency effect, assuming subject compliance (confirmed after each experiment), we can know some latency difference between L- and R-SMC exists, without knowing what that latency actually is. Nevertheless, in cases where a significant difference is found, we additionally tested to see if the identified difference was significantly different than 1 s. Finally, to generate a post-hoc estimate for the difference in Δt (i.e. the relative lag) in the ROIs, we averaged the signals within the chosen ROIs and performed a final fit.

## Results

### Simulation results

**Fig. 3:**
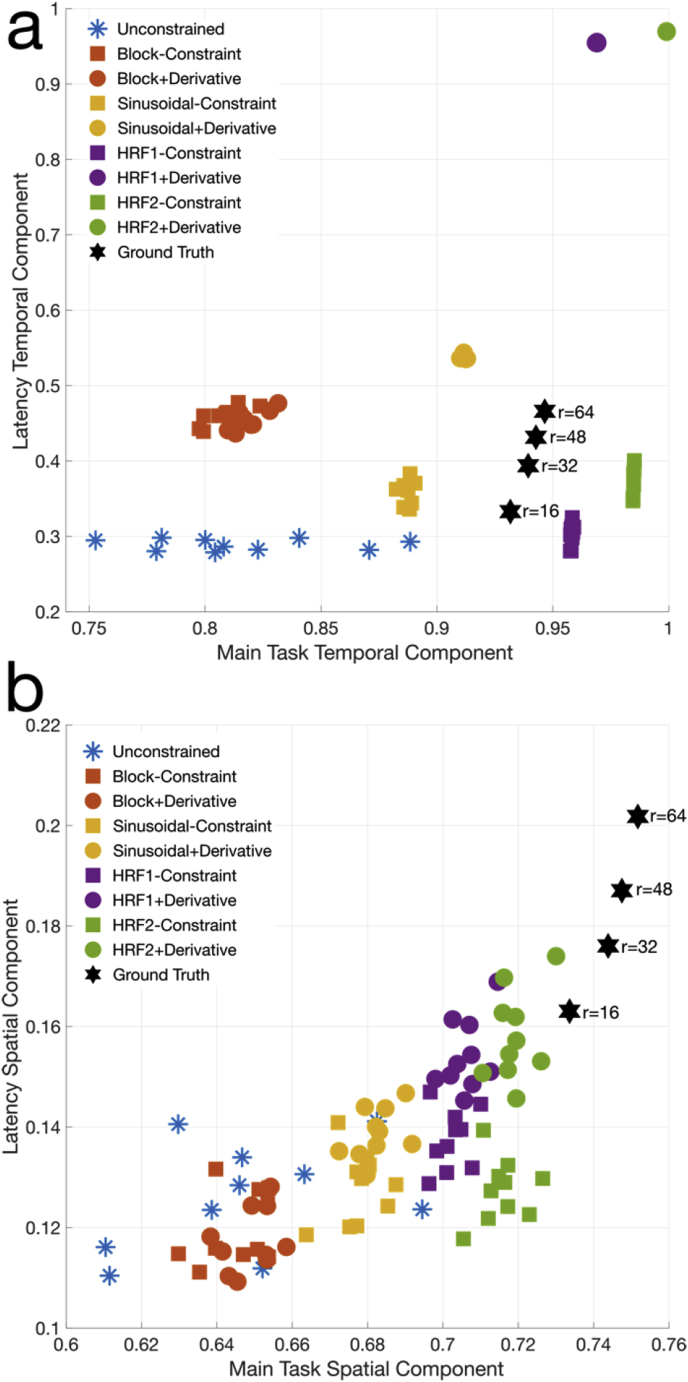
Temporal and spatial subspace fidelity measured by canonical correlation, across 10 simulation repeats. The proposed method with task only, and task and derivative constraints was compared to an unconstrained reconstruction, and a fully-sampled ground truth with the same additive noise. (a) Temporal correlations and (b) spatial correlations of the estimated rank-16 subspaces. The low CNR of the ground truth data, truncated at rank 16, 32, 48 and 64 for illustration, is why perfect correlations are not achieved.

First, we assessed how well the total reconstructed subspaces captured the temporal and spatial characteristics of the simulation components, which can be seen in Fig. 2b. This was measured by looking at the angle between the vectorized representation of the temporal or spatial signals and the reconstructed subspace, or equivalently, by examining the signal-to-subspace canonical correlations. Fig. 3 shows the results of all 10 simulation repeats, across reconstructions using only the different task models given in Fig 1b as constraint, and using both task and derivative models. To illustrate that the simulation was performed in a relatively low CNR regime, near the detection limit, the canonical correlations for the noisy, but fully-sampled equivalent are additionally shown at different truncated dimensionalities. As expected, the more informative constraints produce better correlations. Because the temporal constraints are included in the estimated temporal subspace by design, we see temporal correlations very close to 1 for the HRF1 and HRF2 models (Fig. 3a). More importantly, the spatial correlations also show improvement with increasing fidelity of the temporal constraint (Fig. 3b), illustrating the improvement in quality of reconstructed spatial information, despite the fact that no spatial constraints were applied. The HRF1 and HRF2 constraints bring us close to the spatial fidelity achieved with fully sampled data, meaning that the limiting factor is noise, and the effects of under-sampling are largely mitigated.

**Fig. 4:**
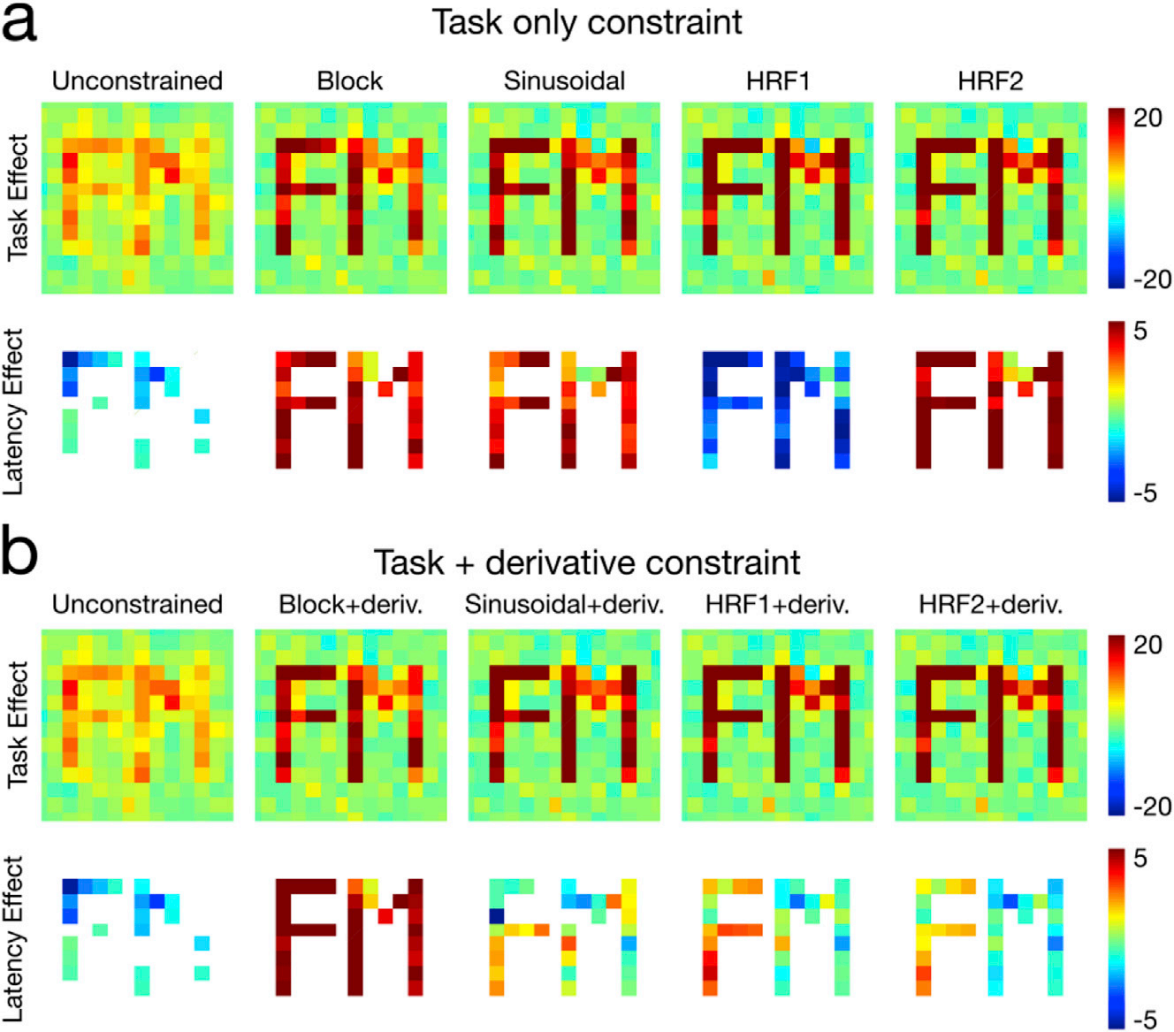
Spatial z-statistic maps of the task and latency components in the simulated reconstructions with various temporal constraints, compared to an unconstrained reconstruction. These are zoomed and cropped over the ROIs for clarity.(a) Using only the task waveform constraint shows good recovery of the spatial ROIs associated with the main task, but no discrimination in latency between the ROIs. (b) Using both task and temporal derivative constraints, we observe identical task component recovery, but also improved sensitivity of the polarity differences in the ROI latencies, particularly in the HRF1 and HRF2 constraints.

Looking specifically at the spatial characteristics of the simulated reconstruction, we see the same pattern manifesting in the z-stat maps generated by directly evaluating the standard error of the spatial parameter estimates associated with the temporal constraint across the 10 simulation repeats (Fig. 4). We see in Fig. 4a the spatial maps generated using the task constraint only, comparing the unconstrained reconstruction with the various constraint models. While the task contrast (both ROIs positive) is clearly delineated, no relative latency is apparent in the estimates (ROIs are the same polarity). Fig. 4b shows the results with the inclusion of the temporal derivative, which shows a positive/negative polarity difference between ROIs (“F” vs “M”) with the HRF1/HRF2 constraints, and to a lesser extent the sinusoidal constraint. Notably, the unconstrained reconstruction, generated only with the low-rank model, is not able to identify the subtle latency differences in the data (i.e. both “F” and “M” are in the blue color map), and the reconstructions using the block constraint show strong, undifferentiated latency response related to the lack of haemodynamic delay in the block design waveform. While simulations near the detection limit in this low-CNR regime clearly show the benefit of the constrained approach over the unconstrained low-rank reconstruction, an additional simulation at high CNR (Supplementary Data) shows that the lack of latency effect in the unconstrained reconstruction is not a fundamental limitation, but sensitivity-dependent effect.

**Fig. 5:**
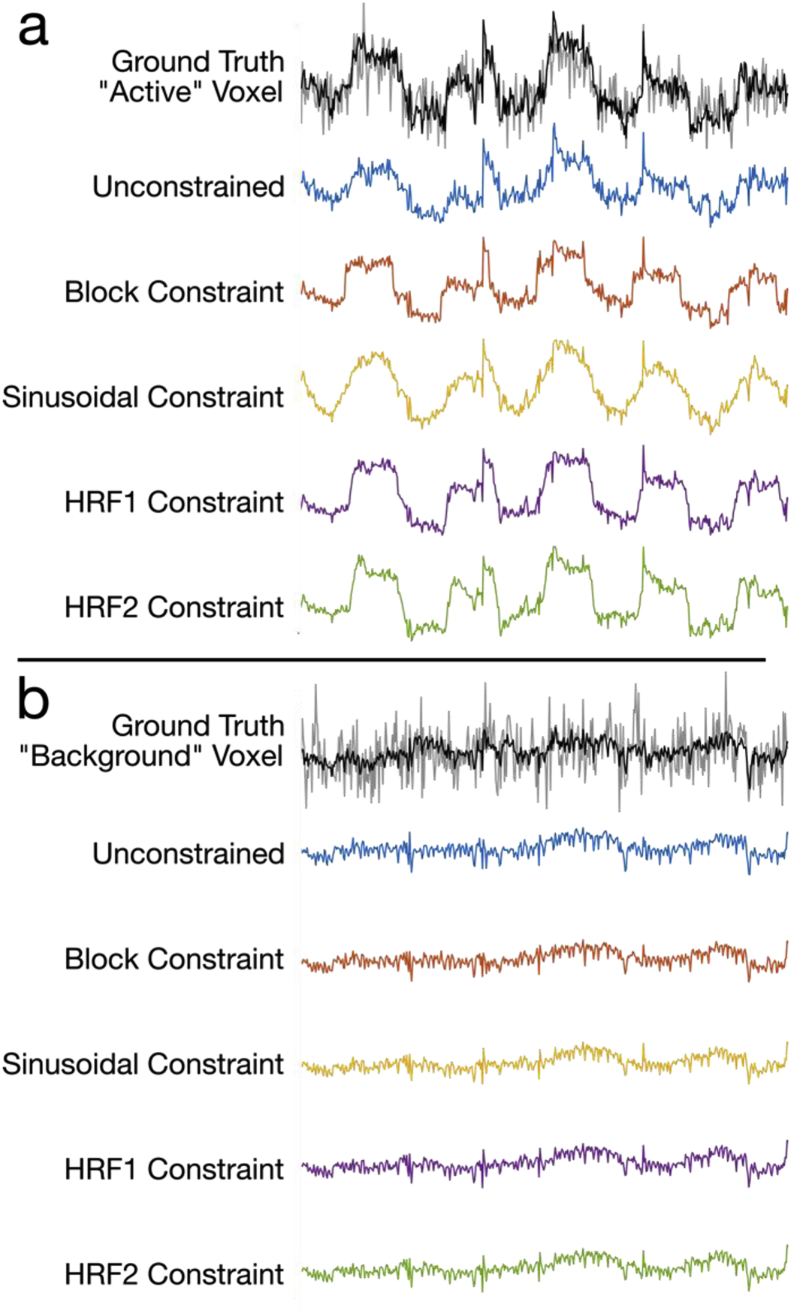
Example time-courses from the reconstructed data from (a) a voxel in the “F” ROI, and (b) a non-specific voxel in the centre of the brain. The ground truth are shown in grey/black, with the full-rank signal in grey, and the rank-16 truncation in black. Unconstrained, block, sinusoid, HRF1 and HRF2 constraint time-courses are shown in blue, orange, yellow, purple and green respectively.

Signal temporal characteristics are shown for an example voxel in the “F” ROI (Fig. 5a) compared again to the noisy fully-sampled ground truth. The impact of the choice of constraint is visible, with the shape of the reconstructed time-courses in each case bearing a resemblance to the specific task model. Nevertheless, each time-series clearly captures some of the variance contained in the signal that is not directly contained in the corresponding temporal constraint. In a voxel from the centre of the simulated brain (Fig. 5b), we expect no “activation”, and all of the different reconstructions are virtually indistinguishable in that they show virtually no model bias, and the low-rank model fits very little of the random, voxel-specific noise.

**Fig. 6:**
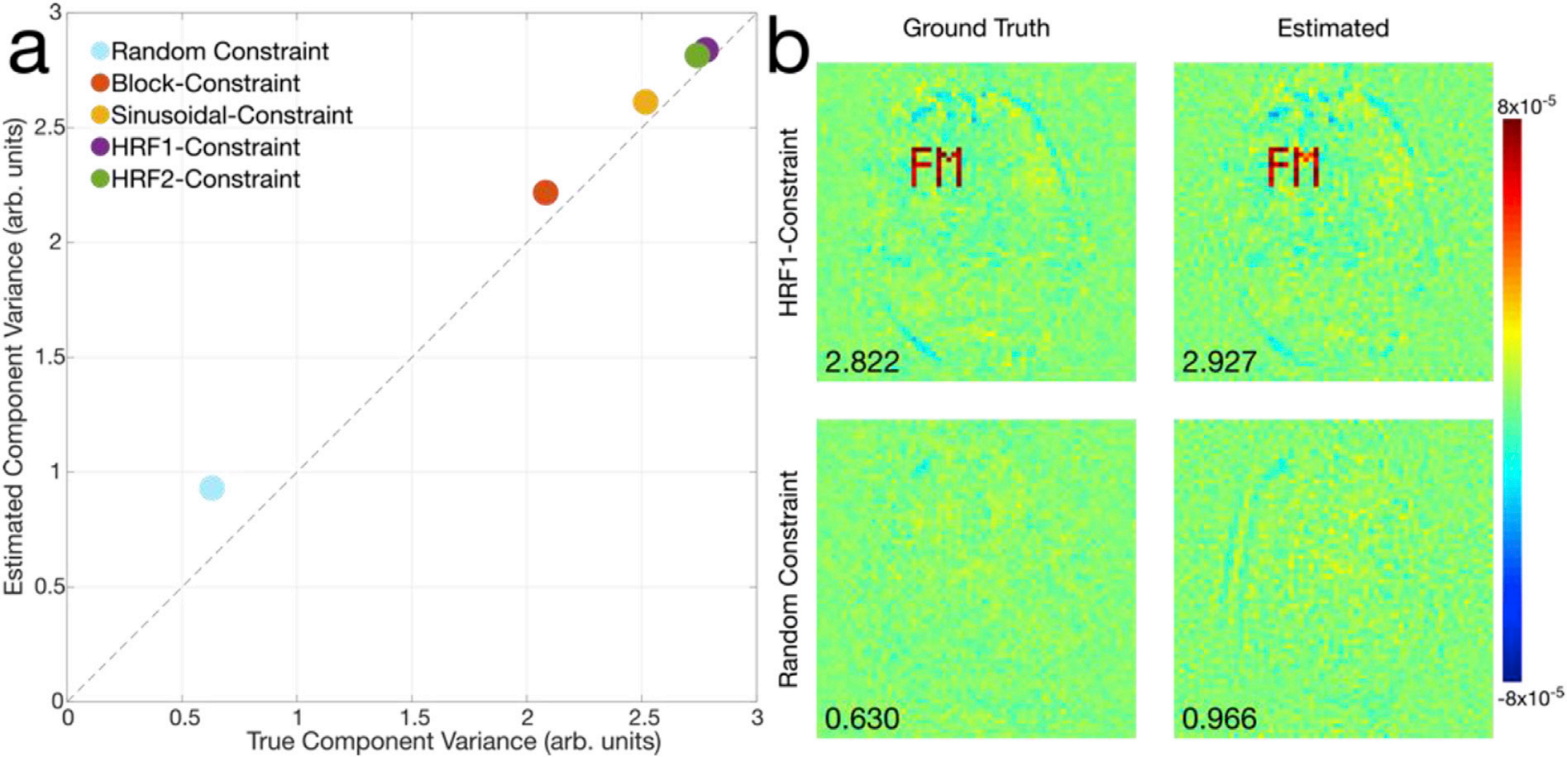
Examination of the variance modelled by the regression/GLM constraints in comparison to the true amount of variance and its spatial distribution. (a) A scatter plot showing the total variance modelled by the various constraints, along with the random constraint. A slight bias is observed, with the values lying above the diagonal, which denotes equality with the ground truth. Markers are larger than the standard deviation of these estimates, across 10 runs. (b) A typical example of the spatial distribution of variance across the reconstructed image (shown as the regression coefficients) for one HRF1 and random-constraint reconstruction compared to the ground truth. In both estimated cases, elevated coefficients can be seen, with the total variance shown in the bottom left.

To quantitatively assess the bias introduced by the proposed approach, we evaluated the variance explained by the task constraint time-courses in the ground truth and reconstructed data. A random Gaussian temporal constraint was also compared to illustrate the case where the constraint is expected to have a low variance contribution and no spatial coherence. In Fig. 6a, we see the total variance associated with each time-course across 10 repeats, which lie slightly above the line of identity, meaning that the proposed reconstructions do slightly bias the data by over-representing its total variance contribution. In Fig. 6b, we can see the spatial distribution of the regression coefficients associated with the HRF1 task constraint, showing elevated background contributions, as well as some bias immediately adjacent to the ROIs. A spatial bias arising from the random constraint is also clearly visible.

**Fig. 7:**
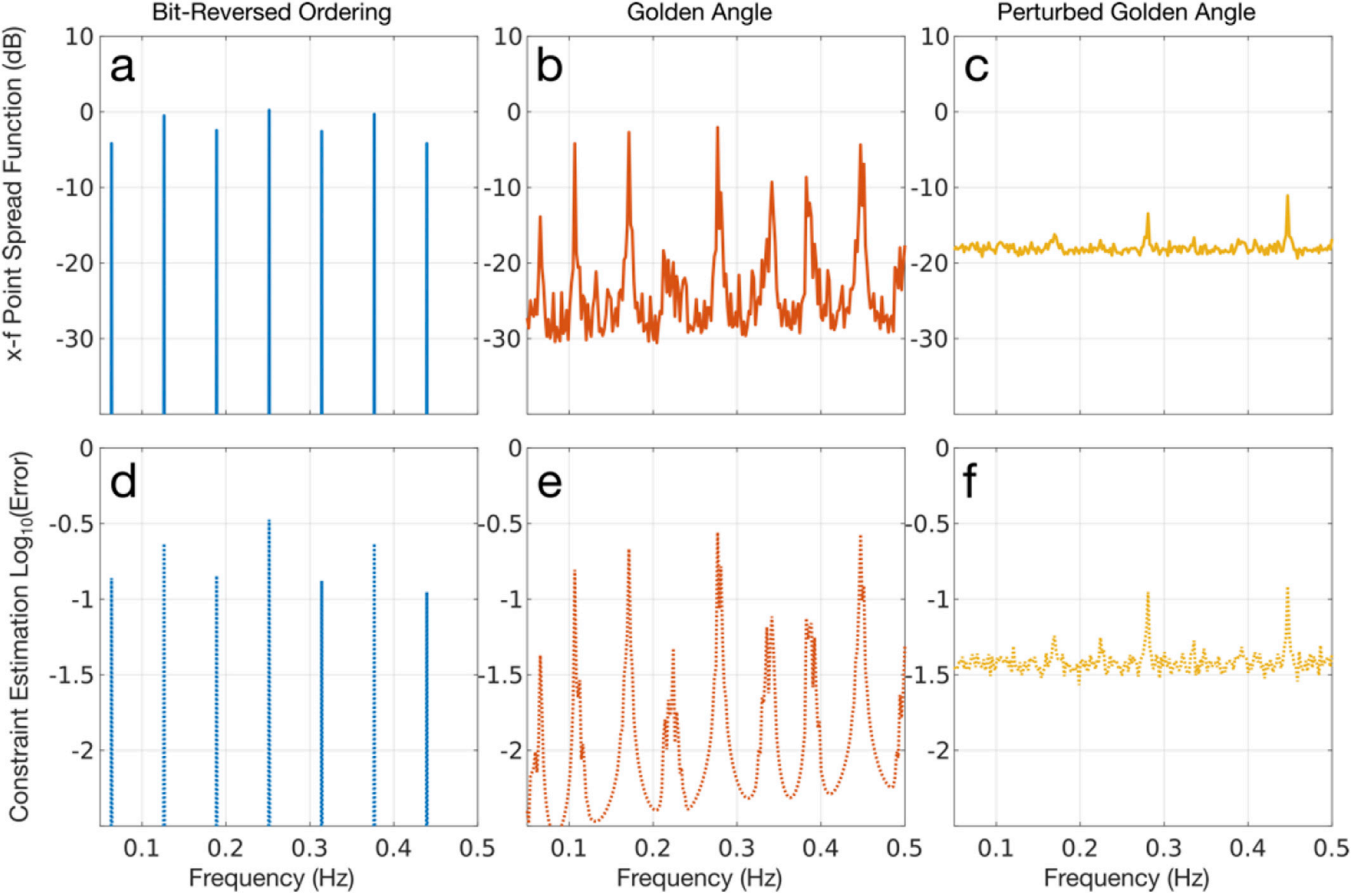
A comparison of regression bias in relation to the sampling PSF, which characterizes residual aliasing. (a-c) Computed x-f PSFs for bit-reversed, golden angle, and perturbed golden angle radial sampling respectively. Here, the PSFs are summed across space to represent the total amount of aliased energy as a function of frequency. (d-f) Estimation error (bias) associated with a constant test object, given input temporal constraints spanning the sampling bandwidth. Peaks in the error estimates clearly coincide with peak side-lobes in the PSFs.

To test whether the elevated bias is due to correlations between the constraint waveform and the sampling PSF, we compared three different radial sampling schemes, with different aliased energy distributions: bit-reversed ordering, which is derived from inverting the binary representations of an ordered set of projections (Chan et al., 2011), conventional golden angle ordering (Winkelmann et al., 2007), and golden angle ordering with a Gaussian perturbation with 5° standard deviation. In Fig. 7a–c, we see the total amount of aliased energy contained in the x-f PSF, summed across all space, showing only the positive half of frequency space. With these samplings, we evaluated the individual impact of pure complex sinusoidal constraint waveforms spanning the positive frequency domain, on a constant test object (that should have no signal energy at non-zero frequencies). The total amount of power of the estimates (i.e. error) associated with each constraint frequency is plotted in Fig. 7d–f. When the frequency of the constraint waveform coincides with a peak side-lobe of the PSF, we find elevated error, which is consistent with the interpretation of bias as a result of PSF effects. We note that the impact of randomly perturbed golden angle sampling is that it has a greatly homogenized aliasing spectral density, with significantly reduced peak side-lobe power, minimizing the maximum possible error for any constraint waveform.

**Fig. 8:**
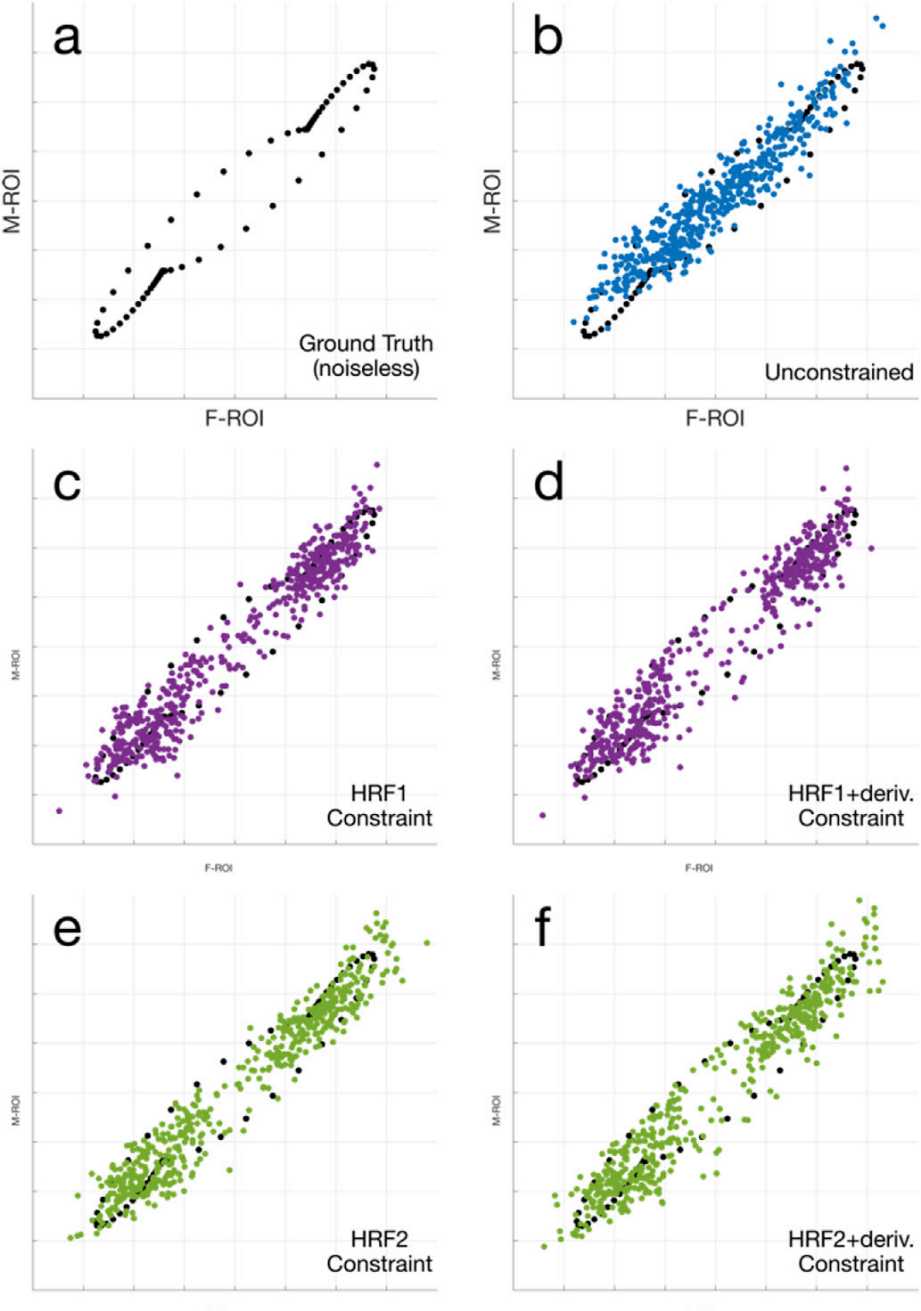
Phase-space scatter plots of data averaged within the “F” and “M” ROIs respectively. (a) Noiseless ground truth signal, which is also displayed underneath (b-f) for visual comparison. (b) Unconstrained reconstruction, (c,d) HRF1 constrained reconstruction, with and without the temporal derivative, (e,f) HRF2 constrained reconstruction, with and without temporal derivative. In (d) and (f), the effect of the derivative constraint in characterizing the latency differences between the ROIs is evident.

Returning to the fidelity of latency estimation in the simulations, we visually assess the lag or phase between ROIs by plotting the signals from the two ROIs against one another to generate a phase space representation of the latencies (Menon et al., 1998). In these representations, signals that have no relative latency will lie along a line, whereas periodic signals that are out of phase will trace out an ellipsoidal where the minor axis scales with the relative latency. Fig. 8 illustrates latency plots generated by averaging over the known ROIs, with the noiseless ground truth signal in Fig. 8a for comparison. Figs. 8d and 8f show the impact of including the temporal derivative constraint, with a wider ellipsoidal shape capturing latency differences, compared to the unconstrained (Fig. 8b) and task-only constraint reconstructions (Fig 8c and 8e).

**Fig. 9:**
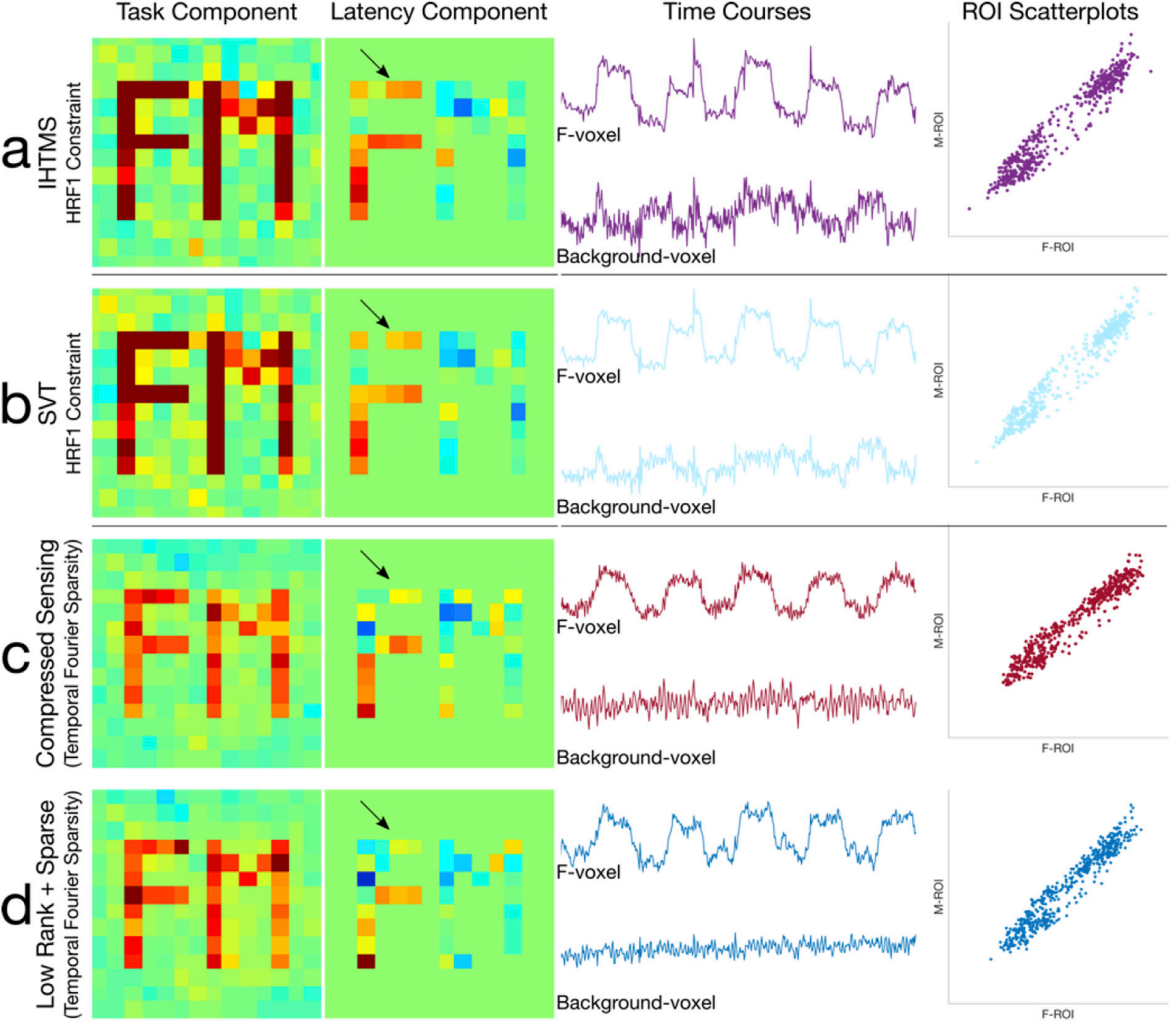
A comparison of the constrained k-t FASTER method (a) with an alternative implementation via the SVT approach (b), as well as a CS reconstruction (c) and L + S reconstruction (d) using temporal frequency sparsity. The first two columns show z-statistic images for the task and latency components, followed by representative time-courses and latency phase plots. Reconstructions in (a) and (b) are virtually identical, illustrating that given some equivalent $\lambda_{SVT}$, the IHTMS and SVT methods perform similarly. In (c) and (d), however, the latency component contains considerable heterogeneity, particularly in the lack of positive latency in the upper portion of the "F" ROI (arrows). The IHTMS and SVT constrained reconstructions show more specific variance being captured (cf. the ground truth voxel in Fig. 5a), whereas the CS and L + S time-courses appear more biased towards the sparse representation. Better latency differentiation in the constrained low-rank approaches compared to the CS and L + S approaches, which is also evident in the phase-space scatterplots.

The proposed constrained k-t FASTER approach using the IHTMS algorithm was compared with an equivalent SVT reconstruction algorithm, both using the HRF1 constraint, alongside a temporal frequency sparsity CS and L + S reconstructions in Fig. 9. We show that with careful choice of $\lambda_{SVT}$, the IHTMS and SVT results are virtually indistinguishable, across spatial and temporal metrics. The CS and L + S produced similar results, with more heterogeneous z-statistics estimates and biased time-courses. Resulting normalized root mean square errors for the various methods are 3.61% (IHTMS), 3.36% (SVT), 3.62% (CS) and 3.58% (L + S) respectively. For simplicity, we evaluated the CS method only in the Experimental data as representative of sparsity-driven reconstructions.

### Experimental results

**Fig. 10:**
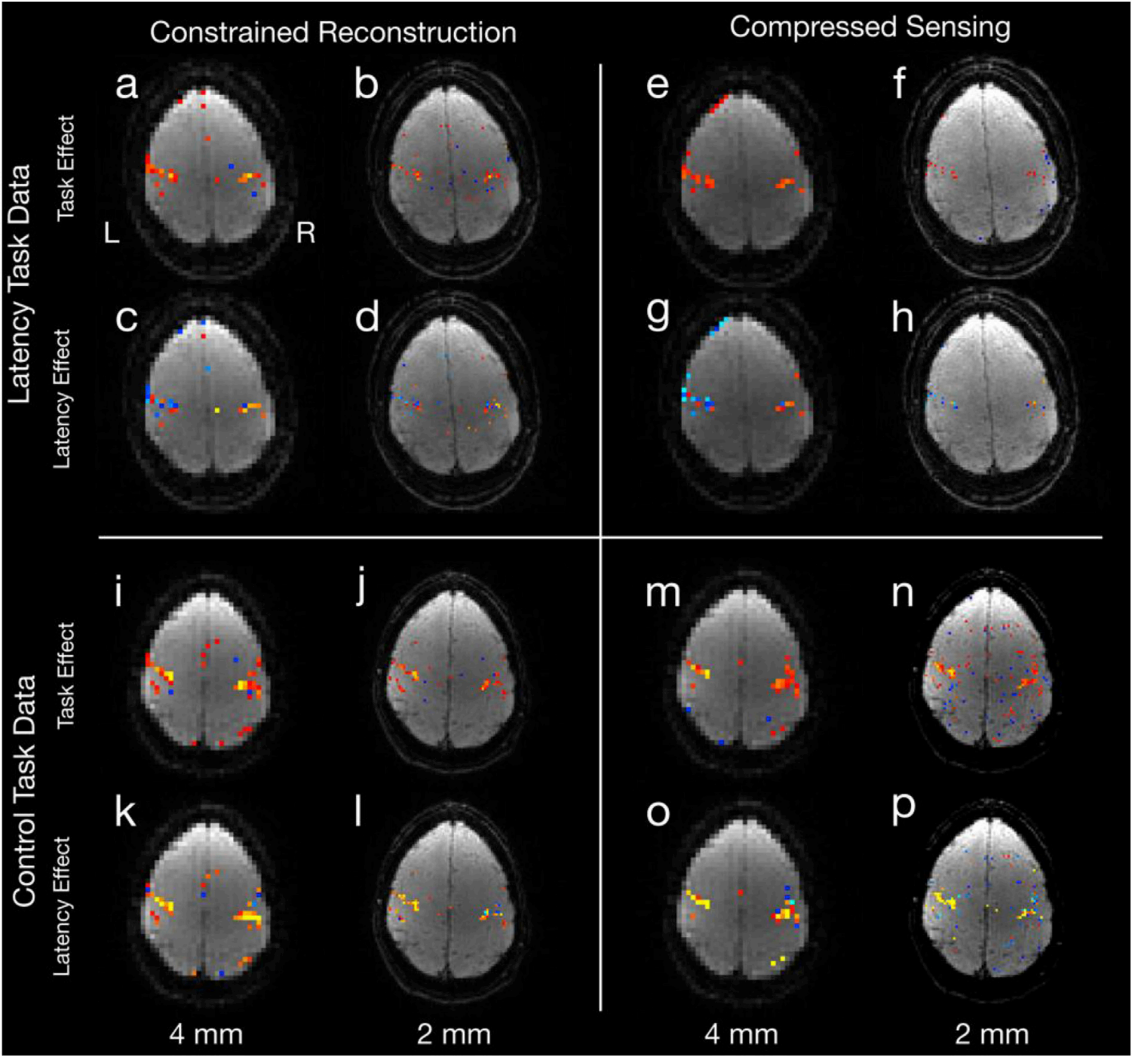
Reconstructed z-statistic images for the 2 mm experiments, showing the latency finger tapping task in the upper quadrants (a-h), and the control (finger tapping with no latency) task in the lower quadrants (i-p). The left quadrants (a-d, i-l) show the constrained reconstruction (using the HRF1 constraint and temporal derivative), and the right quadrants (e-h, m-p) show the CS reconstruction ($\lambda_{CS}=1.58\times 10^{-5}$). Within each quadrant, the top row (a,b,e,f,i,j,m,n) shows the task z-statistic and the bottom row shows the latency (c,d,g,h,k,l,o,p), masked by the task. The left-most columns of each quadrant (a,c,e,g,i,k,m,o) show a 4 mm reconstruction of the same data, with the nominal resolution 2 mm reconstruction on the right (b,d,f,h,j,l,n,p). Task images are all thresholded at (3 < |z| < 10), and latency images are shown on a (|z| < 3) scale, masked by the task response. All z-statistics are overlaid on example reconstructed images.

Results from the functional task experiment demonstrate the ability for the constrained k-t FASTER approach to capture subtle latency differences at considerable acceleration factors. In Fig. 10, results for the 2 mm data are shown reconstructed at the nominal 2 mm resolution (R = 10), and at a reduced resolution of 4 mm (R = 5), where the latter provides an estimate closer to the ground truth by reducing the under-sampling burden. In all cases, there is a robust response of the main task effect in both L- and R-SMC. As expected in the latency experiment, we also see negative (blue) latency coefficients in the L-SMC and positive (red) in the R-SMC for both methods (Fig. 10d, 10h), which is particularly clear in the 4 mm data (Fig 10c, 10g). In the control experiment, while the task responses were equally strong, no positive/negative latency difference is evident in the z-statistic maps. The CS approach shows similar z-statistic maps.

The upper half of Table 1 summarizes the statistical significance of the latency estimations across these datasets which largely mirror the qualitative results in Fig. 10. Here, given that the latency estimates are generated by averaging the signal over the ROIs defined by main task activation, we would expect similar values between the 4 mm and 2 mm reconstructions. In the latency experimental data, we see significant latency estimates ranging from 1.41 s to 2.19 s for the constrained k-t FASTER and CS approaches. In comparison, the unconstrained reconstruction was unable to reject the null hypothesis (that there is no latency in response between left and right motor cortices) in either case. We also found that only the 4 mm reconstruction showed a latency significantly different from 1 s, although given that this occurred in both the constrained k-t FASTER and CS reconstructions, it could be the result of a true latency greater than 1 s. The 2 mm latency data were close to significance in both cases as well, with p-values of 0.11 and 0.06 respectively, and this could reflect the fact that 2 mm latencies were under-estimated relative to the 4 mm reconstructions. In the control data, we expect a null result, as no experimental manipulation has occurred, but we see that in this case, the CS reconstruction falsely rejected the null hypothesis, likely due to signal bias from the sparse representations.

**Table 1:** Estimated latencies and p-values for these comparisons, with significant estimates (thresholded at p < 0.05) indicated in bold, with an asterisk. The p1 values refer to the "any latency difference" test, and the p2 values refer to the test of whether latency means were 1 s apart.

Task	Dataset	Constrained k-t FASTER	CS	Unconstrained
Latency	4.0 mm (Subject 1)	2.19 s **p1** = 0.0083* p2 = 0.041*	1.76 s **p1** = 0.0019* p2 = 0.011*	0.26 s p1 = 0.17 (ns) p2 = –
	2.0 mm (Subject 1)	1.41 s **p1** = 0.025* p2 = 0.11 (ns)	1.51 s **p1** = 0.0027* p2 = 0.058 (ns)	0.63 s p1 = 0.096 (ns) p2 = –
	1.5 mm (Subject 2)	1.07 s **p1** = 0.038* p2 = 0.080 (ns)	0.72 s p1 = 0.42 (ns) p2 = –	0.46 s p1 = 0.13 (ns) p2 = –
	1.5 mm (Subject 3)	1.70 s **p1** = 0.021* p2 = 0.11 (ns)	1.58 s **p1** = 0.018* p2 = 0.077 (ns)	−0.05 s p1 = 0.52 p2 = –
No Latency	4.0 mm (Subject 1)	−0.51 s p1 = 0.62 (ns)	−0.98 s p1 = 0.23 (ns)	−0.35 s p1 = 1.00 (ns)
	2.0 mm (Subject 1)	−0.58 s p1 = 0.44 (ns)	**−0.94s** **p1** = 0.0057*	−0.35 s p1 = 0.25 (ns)
	1.5 mm (Subject 2) (control ROI)	73.56 s p1 = 0.79 (ns)	−5.89 s p1 = 0.48 (ns)	−0.25 p1 = 0.20 (ns)
	1.5 mm (Subject 3) (control ROI)	−7.38 s p1 = 0.095 (ns)	13.38 s p1 = 0.12 (ns)	1.60 s p1 = 0.59 (ns)

**Fig. 11:**
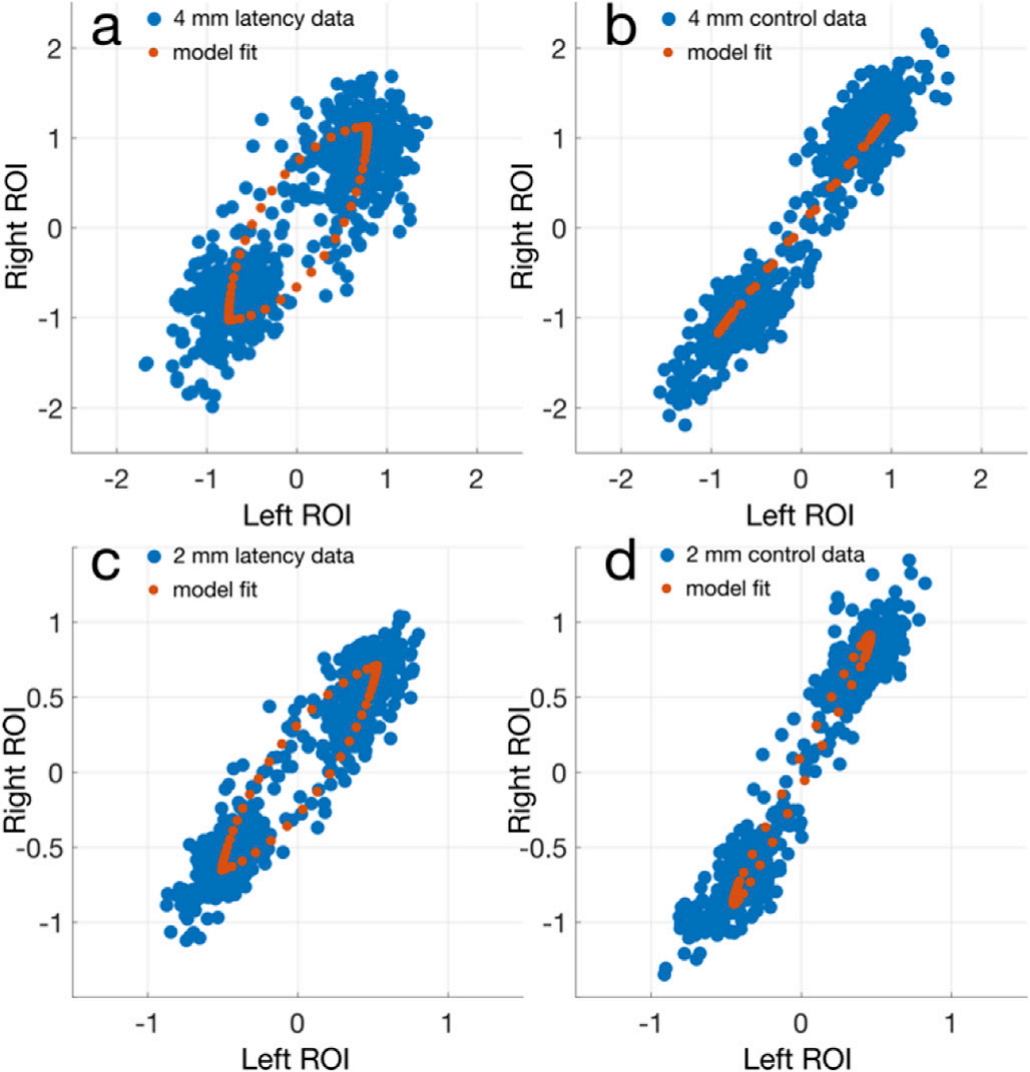
Phase space scatter plots for the 2 mm experiments, with the GLM model fit over the averaged ROI response overlaid. (a,c) the latency experiment data, reconstructed at 4 mm and 2 mm respectively, and (b,d) the control data reconstructed at 4 mm and 2 mm. The qualitative difference in the latency data compared to the control data highlight the sensitivity and specificity of the proposed method.

**Fig. 12:**
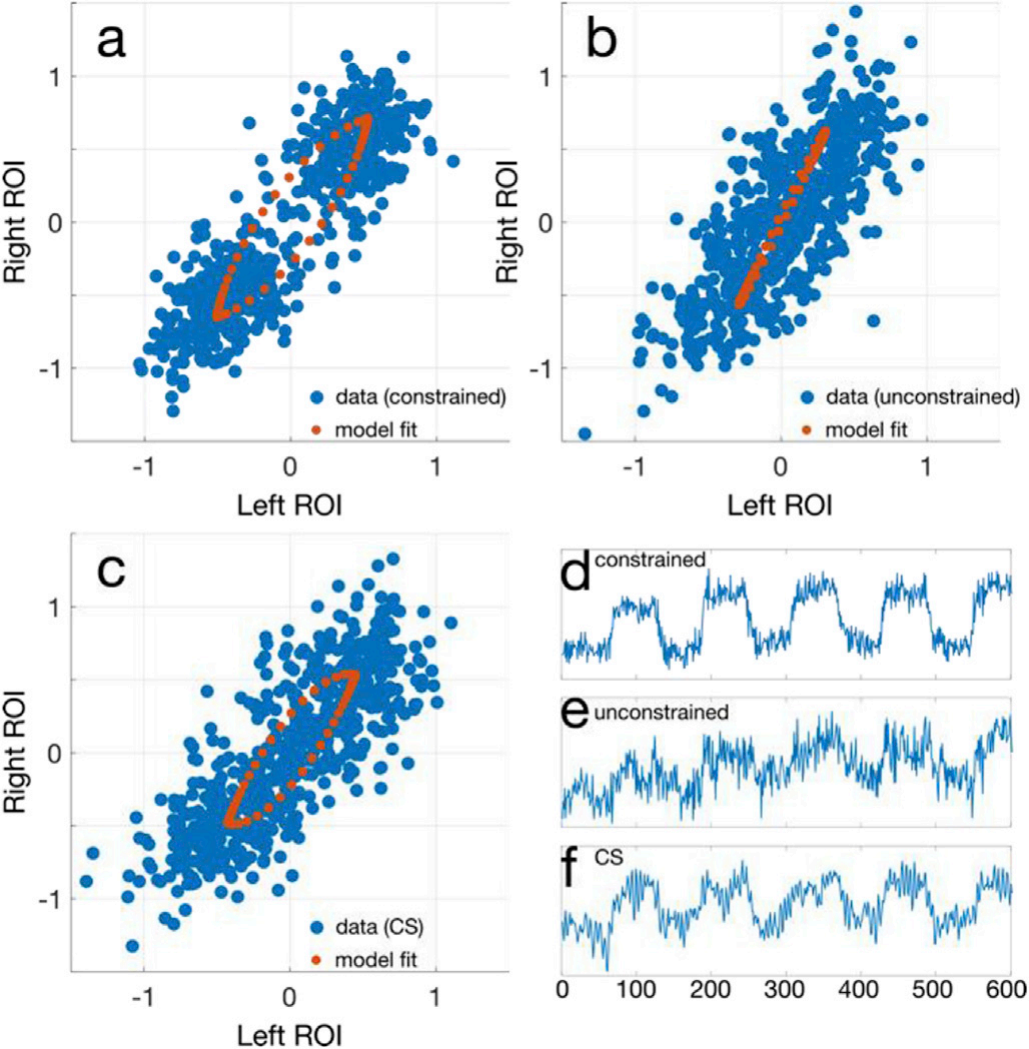
Phase space scatter plots for the 2 mm latency experiment, with the HRF1 model fit overlaid. (a) The constrained reconstruction is compared to the (b) unconstrained reconstruction, and (c) the CS reconstruction. Also shown are averaged voxel time-courses over the left ROI for the (d) constrained, (e) unconstrained, and (f) CS data.

Fig. 11, Fig. 12 show the latency phase-space plots using the same ROIs for the 2 mm constrained reconstruction, with Fig. 11 highlighting the visible differences between the latency and control experiments, in both 4 mm and 2 mm reconstructions in the proposed approach. In Fig. 12, the difference between the proposed constrained, unconstrained, and CS reconstructions in the 2 mm latency experiment are shown, with ROI averaged time-courses in Fig 12d–f.

Table 1 also summarizes the results from both subjects in the 1.5 mm experiments, along with latency estimates from control ROIs defined by a 10 voxel shift in the anterior direction. Here, the results mirror the 2 mm data, where the proposed method the was able to reject the null in both subjects, whereas the CS reconstruction failed to achieve significance in subject 2. Both methods did not reject the null in the control ROIs, and the unconstrained reconstructions similarly failed to reject the null, as expected.

## Discussion

We have demonstrated the feasibility of using temporal constraints derived from experimental design information to facilitate image reconstruction of highly under-sampled fMRI data. In simulations and experiments, we show that the proposed method enables recovery of subtle and low-CNR spatio-temporal features like relative latencies in the BOLD response between comparable cortical regions, even when no spatial information is used (or needed) to constrain the reconstruction. In our previous work using only the low-rank model for reconstruction (Chiew et al., 2015; Chiew et al., 2016), we have shown that recovery fidelity of functional components is related to the strength (relative variance) of the components. As represented here by the “unconstrained” reconstruction, relatively weak effects are not well captured, whereas extending the low-rank approach with incorporation of the temporal constraint greatly improves recovery fidelity, although this requires prior knowledge of the expected signals. Here, we were able to generate whole-brain functional images at isotropic resolutions up to 1.5 mm, while retaining volume TRs ≤600 ms, with TEs affording optimal BOLD contrast. These data, reconstructed at acceleration factors from R = 10 to 16, retained sensitivity to subtle features of the BOLD response in the latency task tested here. This approach could be a useful alternative for ultra-high resolution fMRI, such as for layer-specific imaging (Koopmans et al., 2011), as we have shown that only small amounts of effective spatial resolution loss can be expected using low-rank constraints, even at high under-sampling factors (Chiew et al., 2016).

Crucially, this GLM + PCA-inspired reconstruction produces sensible data reconstructions at under-sampling factors that result in very ill conditioned image reconstruction problems, even with the use of coil sensitivity information. We demonstrated this using 3D radial-Cartesian sampling, but the constrained k-t FASTER approach is compatible with any suitably incoherent k-t sampling pattern, such as CAIPI-sampled 3D-EPI (Poser et al., 2013) and SMS-EPI by introducing time-varying sampling patterns (Chiew et al., 2017a). The sampling incoherence is an important factor, however, as it controls the level of bias (i.e. false positive results) introduced by the constraint. While the bias is small for the sampling strategy used here, we additionally corrected for it by performing mixture modelling on the statistical parametric maps to correct the null of the z-statistic distributions to correspond to zero mean and unit variance Gaussians, which fit this data well. Mixture modelling is also useful for correcting for bias that results in constrained reconstructions due to implicit noise filtering and/or reduced temporal degrees of freedom.

Here, CS leveraging sparsity in the temporal frequency domain performed nearly as well as the proposed method, illustrating the power of sparse regularization. However, the CS method demonstrated both false positives and false negatives (Table 1): not identifying one out of four true latencies, and falsely finding latency in one of the four control experiments. By comparison, the constrained k-t FASTER reconstruction had no false positives or negatives out of the eight cases. Furthermore, it performed less robustly than the proposed methods in the low CNR simulations in latency identification. While we chose to focus on relative BOLD latency for our block-design experiments, one significant advantage of the proposed approach is that applications are not restricted to block design experiments. For example, the proposed approach could be applied to fast event-related fMRI experiments, where optimal design efficiency requires jittered or randomized trial timings (Dale, 1999), although the constraint model would be more complex to account for inter-event variability. Whereas enforcing sparsity in the temporal frequency domain tends to favor periodic experimental designs, leveraging sparsity is not mutually exclusive with the proposed temporal constraint, and future work may combine low-rank, sparse and explicit temporal constraints for further benefit.

The proposed method bears similarity to methods described in the context of functional imaging analysis, such as semi-blind or regularized ICA methods using temporal constraints (Calhoun et al., 2005), spatial constraints (Valente et al., 2009; Lin et al., 2010), or both (Rasheed et al., 2009; Wang et al., 2014). In these methods, prior information is injected to the ICA process to improve the identification of functional components. Similarly, our proposed approach aims to use temporal information equivalent to a GLM design matrix to improve the estimation of spatio-temporal subspaces that characterize our signals of interest. However, the main difference is that in our case, this information is used to regularize the image reconstruction problem, rather than as an analysis tool after the functional images are formed.

This distinction is of great importance in the presence of under-sampled data acquisition. In fully-sampled acquisitions, there is a unique mapping between the k-space data and the image that is maximally consistent with those measurements, so it is conceptually identical whether the GLM model is fit to the k-space data, or the generated images. However, by using this information as part of the image formation process, and to constrain the output images in conjunction with a low-dimensional model to describe the non-explicitly modelled variance, we are able to identify features of the data that would be otherwise undetectable using either part of the decomposition model alone. This pairing facilitates reconstructions with imperfect knowledge (e.g. by using canonical HRFs), in which the low-rank/PCA part of the reconstruction can describe the unmodelled, but important signal variance. As shown in the simulation results, reconstructions using crude pure sinusoidal constraints or constraints derived from different HRF models were still able to capture the signals of interest, despite not providing perfect information. Furthermore, completely uninformative (e.g. random) constraints only affect the reconstructed data through bias from the sampling PSF and wasted degrees of freedom.

We have only a limited number of total degrees of freedom available due to the under-sampling, so we cannot simply fit a complete temporal basis using this procedure. While there are benefits to both GLM-like models, which make use of what we know, and PCA-like models, which adapt to the variance contained in the data, the advantage of the proposed constrained k-t FASTER approach is in leveraging the strength of both in a way that makes an efficient use of the available sampling degrees of freedom. Additionally, whereas in a GLM, unmodelled signals (e.g. physiological noise) only impacts statistics, in the reconstruction problem unmodelled or uncharacterized variance can lead to misattributed signal (i.e. image artefact) when filtered through the sampling point-spread function. Allowing the low-rank component to capture signal variation in addition to the GLM-like temporal constraint is important in ensuring the robustness of the final estimates.

Data pre-processing pipelines that typically follow image formation remove confounds and generally correct the representation of the data. In our proposed method, we rely largely on the data obeying low-rank assumptions, as the GLM constraints are enforced while images are being formed, without any pre-processing. However, as image reconstruction techniques are becoming increasingly sophisticated, we will be able to perform many, if not all of traditional pre-processing by incorporating these corrections into the measurement model. For example, motion correction can be enabled by estimating and correcting raw k-space (Graedel et al., 2017), physiological noise and nuisance removal can be performed using the approach described below, distortion and off-resonance correction can be formulated as with image reconstruction as a general linear inverse problem (Wilm et al., 2011), and spatial smoothing can be performed more optimally by manipulating k-space sampling trajectories (Kasper et al., 2014).

This approach also presents a more general framework for incorporating any known signals into the reconstructed data subspaces, not just those derived from a task design matrix. For example, this could potentially be used for multi-modality integration, where neuronal fluctuations measured with simultaneously acquired electroencephalography (EEG) could be transformed and used to constrain the fMRI temporal subspace, on the assumption that shared variance exists between the modalities. This type of external information could allow the constrained k-t FASTER method to be applied not just in task-fMRI, where the neuronal manipulation is known *a priori*, but also to resting state conditions where EEG signals are instead used to predict resting state signal variance (Chiew et al., 2017b). Other possible sources of temporal information include externally acquired physiological confound traces (e.g. respiratory, cardiac), which could be used to regress out physiological nuisance effects when explicitly modelled in the reconstruction. Furthermore, this is also not restricted to temporal constraints, and a similar procedure can be used to integrate spatial prior information, such as *a priori* functional parcels (Wong, 2014).

Although it may appear that the design matrix is used twice, to reconstruct the data and then subsequently to generate spatial z-statistic maps, in fact, the information in the design matrix is being used in the same way, multiple times for practical convenience. In a very real sense, incorporating the GLM constraint in the reconstruction model directly estimates the GLM regression coefficients as a part of the (complex) image formation process. However, after magnitude transform of the image data, and detrending or filtering, the coefficients need to be re-estimated in a final fit, analogously to what would be done in a conventional analysis. Furthermore, this approach does not require that a GLM analysis be performed at all, where the reconstructed data could just as easily be analyzed by model-free methods.

## Conclusion

We have presented a new method for constrained image reconstruction of highly under-sampled fMRI data, by leveraging information from GLM experimental design matrices as part of the image reconstruction process, in conjunction with low-rank modelling. This approach is compatible with many of the other methods used for efficient sampling of fMRI data, including compressed sensing, and parallel imaging in 3D and SMS-EPI, and could be used in future to facilitate even larger gains in sampling efficiency. The framework presented also permits other extrinsic sources of information to be leveraged for highly under-sampled image reconstruction.

## Appendix

### Appendix A SVT algorithm

Initialize $M^{0}$ (e.g., as zeros), step size t, rank r, singular value threshold. λ

Loop until converged:

$$Y^{\left(i+1\right)}=M^{\left(i\right)}+tE^{*}\left(d-EM^{\left(i\right)}\right)$$

$$U^{\left(i+1\right)}=Y^{\left(i+1\right)}V_{c}{\left(V_{c}^{*}V_{c}\right)}^{-1}$$

$$X^{\left(i+1\right)}=shrink_{\lambda t}\left(Y^{\left(i+1\right)}-U^{\left(i+1\right)}V_{c}^{*}\right)$$

$$M^{\left(i+1\right)}=X^{\left(i+1\right)}+U_{c}^{\left(i+1\right)}V_{c}^{*}$$

where

$$shrink_{\lambda t}\left(X\right):\text{max}(diag\left(s_{j}-\lambda t\right),0)$$

and $s_{j}$ are the singular values of. X

The SVT-based algorithm differs from Algorithm 1 only in the way the shrinkage of singular values is applied. Here, the shrinkage is dictated by the parameter λ and the step size t.

### Appendix B FISTA algorithm

Initialize $X^{0}$, $Y^{0}$ (e.g., as zeros), step size t, $k^{0}=1$

Loop until converged:

$$X^{\left(i\right)}=F_{t}^{-1}\left(shrink_{\lambda t}\left(F_{t}\left(Y^{\left(i\right)}+tE^{*}\left(d-EY^{\left(i\right)}\right)\right)\right)\right)$$

$$k^{i+1}=\frac{1+\sqrt{1+4k_{i}^{2}}}{2}$$

$$Y^{\left(i+1\right)}=X^{\left(i\right)}+\frac{k_{i+1}-1}{k_{i}}\left(X^{\left(i\right)}-X^{\left(i-1\right)}\right)$$

The FISTA algorithm applied to the space-time dataset, using the sparsifying transform $F_{t}$, which applies the Fourier transform along the temporal dimension.

## Footnotes

Appendix C Supplementary data related to this article can be found at https://doi.org/10.1016/j.neuroimage.2018.02.062.
