# Supplementary material for: Recovering task fMRI signals from highly under-sampled data with low-rank and temporal subspace constraints
Source: Neuroimage. 2018 Jul 1;174:97–110. doi: 10.1016/j.neuroimage.2018.02.062 (PMC5953310; doi:10.1016/j.neuroimage.2018.02.062)
Supplement: Supplementary material [file mmc1.pdf]

Supporting Information

Accelerated reconstruction algorithm

Initialize M^0 (e.g., as zeros), step size t, rank r, shrinkage factor τ, $k_0 = 1$

Loop until converged:

$$Y^{(i+1)} = M^{(i)} + tE^*\left(d - EM^{(i)}\right)$$
$$U^{(i+1)} = Y^{(i+1)}V_c(V_c{}^*V_c)^{-1}$$
$$usv^* = Y^{(i+1)} - U^{(i+1)}V_c{}^*$$
$$X_r{}^{(i+1)} = u\tilde{s}_{r,\tau}v^*$$
$$k_{i+1} = \frac{1 + \sqrt{1 + 4k_i{}^2}}{2}$$
$$M^{(i+1)} = \left(X_r{}^{(i+1)} + U_c{}^{(i+1)}V_c{}^*\right) + \frac{k_{i+1} - 1}{k_i}\left(X_r{}^{(i)} + U_c{}^{(i)}V_c{}^*\right)$$

Algorithm 2 – Accelerated IHTMS

Algorithm 2 differs from Algorithm 1 only in the way $M^{(i+1)}$ is defined as the input to the data consistency gradient step, where $M^{(i+1)}$ is a function of both the current $(i+1)^{th}$ and previous i^{th} iterates, using a momentum-based update to significantly improve convergence. The impact of the accelerated IHTMS algorithm (Algorithm 2) is shown in Fig. S1, which converges approximately 4-5x faster than the unaccelerated version (Algorithm 1), across 10 different runs. A representative run-time comparison between the algorithms found that Algorithm 1 took 88 iterations (6 min 59s) whereas Algorithm 2 took 17 iterations (1 min 16 s) to attain similar convergence. This comparison was performed using a computer with a 3.1 GHz Intel Core i7 processor and 16 GB RAM.

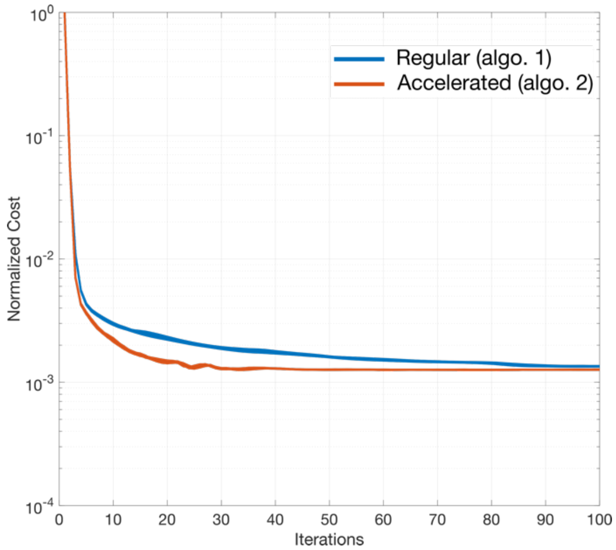

Figure S1 – The benefit of using momentum-based acceleration in the gradient step of the iterative reconstruction algorithm. Ten reconstructions are plotted on top of each other, with the regular (blue) algorithm taking approximately 4-5x longer to converge than the momentum-based acceleration algorithm (b).

High-CNR Simulations

While the low CNR reconstructions in Figs. 3 and 4 highlight the results in a CNR regime near the detection limit, here we additionally show the same reconstructions performed at a higher CNR of 5, to illustrate that the lack of latency detection in the low-CNR unconstrained reconstructions is not a fundamental limitation of the unconstrained low-rank method. Rather, it is a reflection of the fact that signal component fidelity in low-rank reconstructions scale with component strength (i.e. CNR). In Fig S2, we see that in this CNR=5 regime, the task effect of the unconstrained reconstruction is virtually indistinguishable from the HRF2 constrained reconstruction, and the latency effect, although stronger in the HRF2 constrained data, is also robustly present in the unconstrained reconstruction.

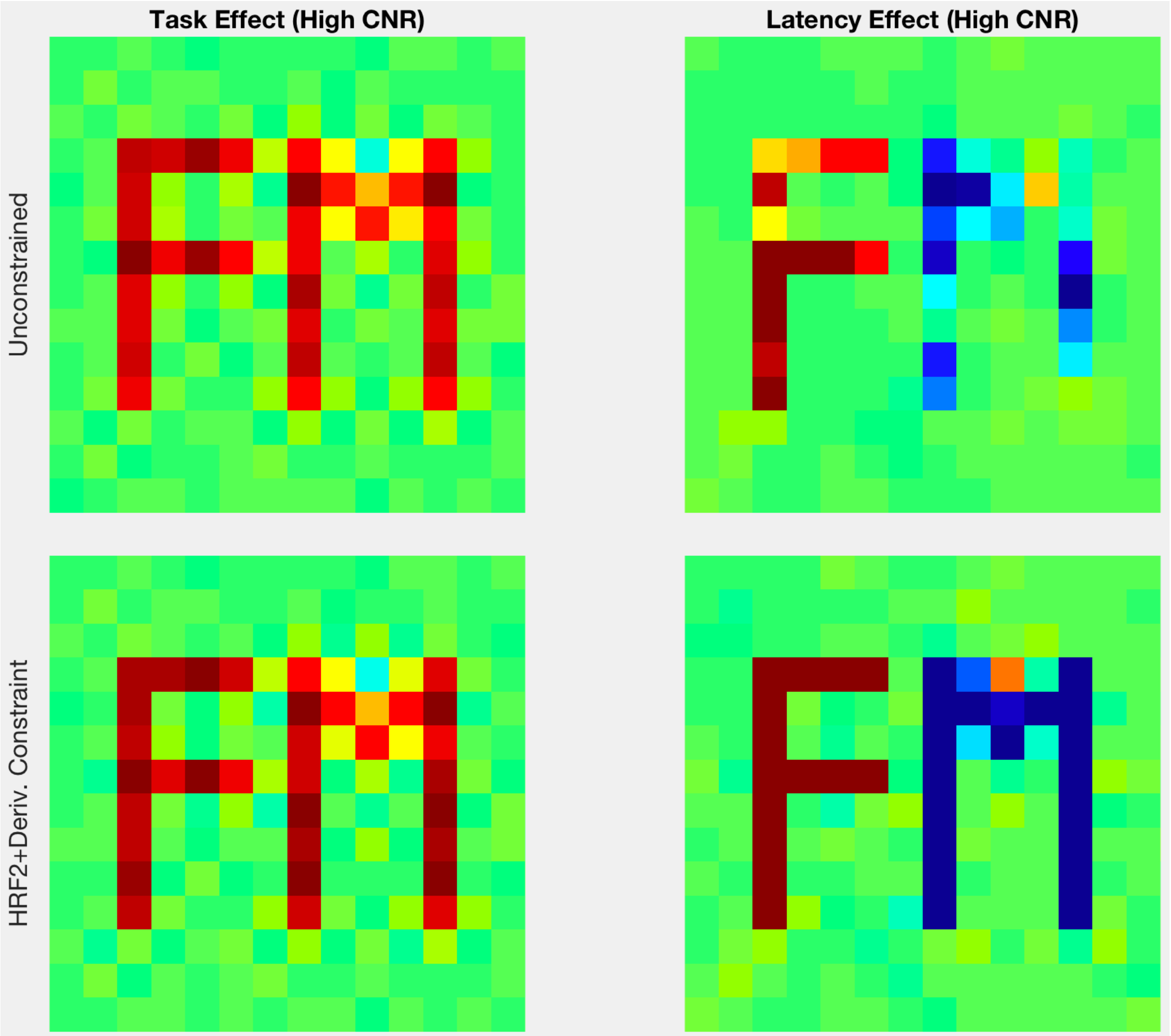

Figure S2 – Results of high CNR simulations of the task and latency effects, in an unconstrained reconstruction compared to an HRF2+temporal derivative constrained reconstruction.

Choice of Rank

We assessed the impact of different choices of the rank cutoff by evaluating the latency z-statistic maps at $r = 2, 4, 8$ and 16, using the HRF1 constraint with temporal derivative.

Here, the rank-2 constraint represents the GLM components only, with no PCA/low-rank residual modelling. The rank 4, 8 and 16 reconstructions on the other hand, contain 2-, 6- and 14-dimensional low-rank models. The impact of the missing low-rank models is evident in the reduced significance of the positive/negative latency coefficients (Fig. S3), which is indicative of the low-rank model absorbing some of the remaining signal variance, which reduces the effective amount of residually aliased signal for the constraint to erroneously model.

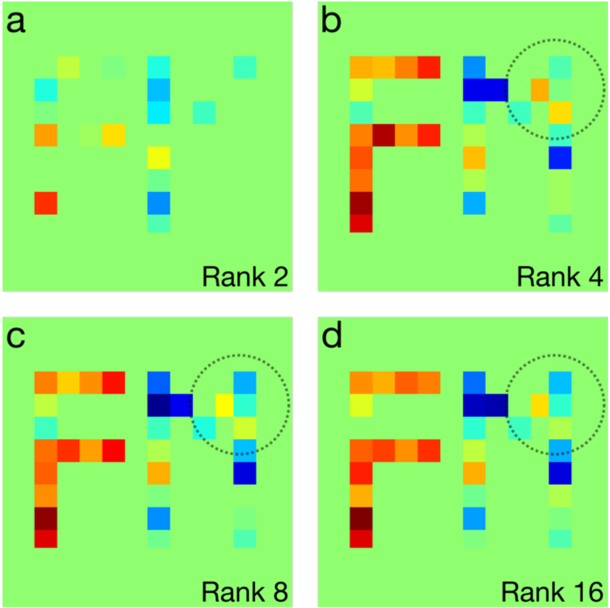

Figure S3 – Comparisons of the spatial fidelity of the latency z-statistic estimates, across a range of choices of the reconstructed rank parameter. The HRF1 and temporal derivative constraints were used. (a) The rank-2 reconstruction corresponds to only these two constraints, and no residual low-rank components. (b-d) Rank 4, 8 and 16 reconstructions, corresponding to rank 2, 6 and 14 low-rank models of the temporal constraint (GLM) residuals. The benefit of the additional capture of variance with the low-rank model is evident, and the dashed circle highlights the additional benefit of higher dimensional models.
